# Long-term treatment with sirolimus for pediatric vascular anomalies

**DOI:** 10.1186/s13023-026-04531-y

**Published:** 2026-08-10

**Authors:** Jueun Park, Hyunhee Kwon, Dae Yeon Kim, Seong Chul Kim, Jung-Man Namgoong, Yu Jeong Cho

**Affiliations:** 1 Division of Pediatric Surgery, Busan Paik Hospital, Inje University College of Medicine, Busan, Korea; 2 Division of Pediatric Surgery, Asan Medical Center, Asan Medical Center Children’s Hospital, University of Ulsan College of Medicine, Seoul, Korea; 3 Department of Surgery, Hanyang University Guri Hospital, Hanyang University College of Medicine, 153 Gyeongchun-ro, Guri-si, Gyeonggi-do Republic of Korea

**Keywords:** Pediatrics, Vascular anomaly, Medical genetics

## Abstract

**Background:**

Although sirolimus has emerged as a safe and effective treatment modality for unresectable vascular anomalies, comprehensive long-term follow-up data remain scarce in the current literature. This study aims to evaluate the long-term clinical experience of pediatric patients with vascular anomalies who received sirolimus treatment over five years.

**Methods:**

We retrospectively analysed 17 pediatric patients with vascular anomalies treated with sirolimus over five years in Asan Medical Center. Lesion volumes were measured via 3D volumetric MRI and normalized to body surface area (BSA). Adverse drug effects and therapeutic responses were periodically assessed. Longitudinal response was modeled using generalized additive mixed-effects models (GAMM). Tapering protocols were initiated after 24 months of stability, and alpelisib switch was considered for sirolimus-refractory cases with documented PIK3CA mutations.

**Results:**

With a median follow-up of 78 months, the overall response rate was 81.3%. GAMM analysis demonstrated a significant non-linear volume reduction (*p* < 0.0001), characterized by a rapid initial response within 12–24 months followed by a sustained plateau. Tapering was successful in 70.6% of patients without disease progression. Three patients with suboptimal responses transitioned to alpelisib; those with sufficient follow-up achieved additional volume reduction within 12 months. Long-term sirolimus use was well-tolerated, with no Grade ≥ 3 adverse events reported.

**Conclusions:**

Sirolimus provides sustained long-term efficacy and safety in pediatric vascular anomalies. The observed treatment plateau suggests that dose tapering is a viable strategy for sustained responders. Furthermore, genotype-guided transition to alpelisib offers an effective alternative for refractory PIK3CA-mutant cases.

## Introduction

Pediatric vascular anomalies (VAs) are a heterogeneous group of conditions ranging from benign hemangiomas to life-threatening malformations [1]. Complex or infiltrating vascular anomalies can cause significant functional impairment and morbidity. Traditional interventions, including surgery and sclerotherapy, often face limitations due to the diffuse nature of these lesions and high recurrence rates [2]. 

The identification of the PI3K/AKT/mTOR signaling pathway as a key driver in VA pathogenesis has revolutionized therapeutic approaches [3]. Sirolimus (rapamycin), an mTOR inhibitor, has subsequently emerged as a standard medical therapy, with clinical trials demonstrating its safety and significant short-term volumetric responses [2, 4–8]. 

Despite these advancements, the existing literature primarily focuses on short-to-intermediate follow-up periods [8–10]. Crucial data regarding long-term durability of response, relapse patterns after cessation, and cumulative safety profiles remain scarce. Furthermore, management strategies for patients exhibiting suboptimal responses to prolonged sirolimus monotherapy are not well-defined.

In 2022, the U.S. Food and Drug Administration granted accelerated approval to alpelisib for adult and pediatric patients aged two years or older with severe manifestations of PIK3CA-related overgrowth spectrum requiring systemic therapy, providing regulatory context for genotype-guided PI3K inhibition in selected refractory cases [11]. This regulatory context for genotype-guided PI3K inhibition and raised important clinical questions regarding optimal transition strategies following inadequate response to sirolimus. This study reports our institutional experience with sirolimus treatment over an extended follow-up period, exceeding five years. We evaluate sustained volumetric responses, long-term safety, and clinical outcomes of transition to alpelisib in sirolimus-refractory cases, aiming to optimize the long-term management algorithm for complex pediatric VAs.

## Methods

### Study design and patient selection

This retrospective cohort study was conducted at Asan Medical Center. We included pediatric patients with complicated vascular anomalies who initiated sirolimus treatment since January 2018 and completed at least 5 years of follow-up. Complicated vascular anomalies were defined as lesions that were unresectable, had failed conventional therapies (sclerotherapy or surgical intervention), or posed significant functional or cosmetic concerns. The study protocol was approved by the Institutional Review Board of Asan Medical Center. [IRB no. 2026 − 0729]

### Treatment protocol

Sirolimus was administered orally at an initial dose of 0.8 mg/m² body surface area (BSA) twice daily. Dosing was adjusted to achieve target trough levels of 8–12 ng/mL. Trough levels were monitored every 2–4 weeks during the initial 3 months, then every 3 months once stable levels were achieved.

Initially, tissue biopsy was not routinely performed as the diagnosis was primarily established through clinical and radiologic evaluation. However, biopsy was indicated in specific cases to exclude malignancy when radiologic features were inconclusive or to facilitate the identification of a molecular target in patients exhibiting a suboptimal response to sirolimus.

In cases of limited response to sirolimus after 24–36 months of treatment, particularly when PIK3CA mutations were identified on tissue biopsy, switching to alpelisib (PI3Kα-specific inhibitor) was considered.

For patients demonstrating sustained treatment response and stable lesion volumes for at least 24 months, medication tapering was initiated. The tapering protocol consisted of reducing from twice-daily to once-daily dosing while maintaining the same single-dose amount, followed by a thrice-weekly regimen. Patients were closely monitored with clinical assessments and imaging during the tapering period.

### Imaging assessment and response evaluation

Magnetic resonance imaging (MRI) was performed at baseline and every 12 months thereafter. Lesion volumes were measured on T2-weighted fat-suppressed sequences by a blinded pediatric surgeon using 3D volumetric analysis. To adjust for pediatric growth, all volumes were normalized to the patient’s body surface area (BSA) at each scan.

Treatment response was defined by the percentage change in normalized volume from baseline. Complete Response (CR) was defined as a reduction ≥ 95%. Partial Response (PR) was subcategorized as Significant (≥ 50%), Moderate (20–50%), or Modest (< 20%) reduction. Progressive Disease (PD) was defined as any volume increase. The overall response rate (ORR) represented the proportion of patients achieving CR or PR at the final follow-up.

### Safety assessment

Adverse events were monitored through clinical assessment at each outpatient visit (every 3 months) and laboratory testing including complete blood count, liver function tests, lipid profile, and renal function tests. Adverse events were graded according to the Common Terminology Criteria for Adverse Events (CTCAE) version 5.0.

### Statistical analysis

Continuous variables were expressed as median (IQR) or mean (SD), and categorical variables as frequencies (%). Longitudinal changes in normalized volumes were analyzed using generalized additive mixed-effects models (GAMM) with random intercepts to account for individual variability and non-linear trajectories.

For patients switching to alpelisib, only MRI data obtained during sirolimus therapy were included in the longitudinal sirolimus-response analysis.

Interaction terms were interpreted cautiously and no adjustments for multiple comparisons were applied, as the subgroup analyses (age at initiation and lesion location) were exploratory in nature. Statistical significance was defined as *p* < 0.05 using R version 4.2.1.

## Results

### Patient demographics and baseline clinical characteristics

A total of 17 patients (9 females, 8 males) with complicated vascular anomalies were included in this study. The median follow-up duration was 78 months (IQR: 73–84 months), with a median sirolimus treatment duration of 42 months (IQR: 37–48 months). The median age at treatment initiation was 57 months (IQR: 13–110 months).

The presenting symptom was a mass in 13 patients (76.5%), airway obstruction in 2 patients (11.8%), and pain in 2 patients (11.8%). Regarding timing of diagnosis, 12 patients (70.6%) were diagnosed prenatally by ultrasound, 2 (11.8%) at birth, and 3 (17.6%) during childhood/adolescence.

The primary lesion locations were head and neck in 9 patients (52.9%), extremities in 6 patients (35.3%), and trunk in 2 patients (11.8%). The most common diagnosis was lymphatic malformation (LM) in 11 patients (64.7%), followed by lymphaticovenous malformation (LVM) in 4 patients (23.5%), venous malformation (VM) in 1 patient (5.9%), and Kaposiform hemangioendothelioma in 1 patient (5.9%).

PIK3CA mutation analysis was performed in 7 patients (41.2%). Among these, mutations were detected in 5 patients, including H1047L (*n* = 1), H1047R (*n* = 1), E545K (*n* = 1), and E542K (*n* = 1), and one blood-detected PIK3CA mutation for which variant annotation was unavailable in the source table. Two patients had negative mutation results, and 10 patients did not undergo genetic testing. Detailed patient characteristics are summarized in Table 1.

**Table 1:** Baseline demographic and clinical characteristics of the study cohort (*N* = 17)

No.	Sex	Symptom	Diagnosis period	Site	diagnosis	Type	Start age (months)	Sirolimus duration (months)	MRI outcome	Current status	Tapering	Cesation of sirolimus	Relapse after cesation	Other interventions	Follow-up (months)	Gene	PIK3CA blood	PIK3CA lesional tissue
																Biopsy		
1	F	Mass	Prenatal	Head and neck	LM	Microcystic	60	50	56% reduction	on alpelisib		yes		sclerotherapy 6 / excision 1	86	Done		H1047L
2	M	Mass	Birth	Trunk	Kaposiform hemangioendothelioma		2	19	100% reduction	ceased		yes		triamcinolon injection 3	88	Not done		
3	M	Mass	Prenatal	Head and neck	LM	Macrocystic	13	48	45% reduction	on alpelisib		yes	yes	sclerotherapy 8 / Excision 1	85	Done	Not detected	H1047R
4	F	Airway obstruction	Prenatal	Head and neck	LM	Macrocystic	10	46	97% reduction	on sirolimus	yes			excision 1	83	Not done		
5	F	Airway obstruction	Prenatal	Head and neck	capillary-LVM	Macrocystic	57	27	90% reduction	on sirolimus	yes			sclerotherapy 13 / Excision 3	84	Done	Not detected	E545K
6	M	Mass	Prenatal	Head and neck	LVM	Macrocystic	9	44	87% reduction	on sirolimus	yes			Excison 1 / sclerotherapy 5	80	Not done		
7	F	Mass	Prenatal	Extremities	LM	Microcystic	129	42	259% increase	on sirolimus	yes			sclerotherapy 6 / excision 4	78	Done	Inappropriate	Inappropriate
8	M	Pain	Birth	Extremities	VM		163	41	412% increase	on sirolimus	yes			sclerotherapy 2	78	Not done		
9	M	Mass	Prenatal	Trunk	LM	Microcystic	141	41	69% reduction	on sirolimus	yes			sclerotherapy 2 / excision 4	77	Done	Not detected	E542K
10	F	Mass	Prenatal	Head and neck	LM	Microcystic	12	42	63% reduction	on sirolimus	yes			sclerotherapy 3	79	Not done		
11	M	Mass	Childhood/adolescence	Extremities	VLM	Microcystic	104	69	11% reduction	on sirolimus	yes			beta blocker / sclerotherapy 2	75	Not done		
12	M	Mass	Prenatal	Head and neck	LM	Microcystic	65	37	12% increase	on sirolimus	yes			sclerotherapy 8	73	Done	Not detected	Not detected
13	F	Mass	Prenatal	Head and neck	LM	Microcystic	29	37	30% reduction	on sirolimus	yes			sclerotherapy 4	74	Not done		
14	F	Mass	Childhood/adolescence	Extremities	LVM	Infiltrative VM	110	63	38% reduction	on sirolimus	yes			sclerotherapy 2	68	Not done		
15	F	Pain	Childhood/adolescence	Extremities	LVM		179	64	60% reduction	on sirolimus	yes			Beta blocker / excision 1	64	Not done		
16	M	Mass	Prenatal	Extremities	LM	Microcystic	22	44		on alpelisib		yes		sclerotherapy 2 / excision 2	62	Done	Detected	
17	F	Mass	Prenatal	Head and neck	LM	Macrocystic	11	64	17% reduction	on sirolimus	No			sclerotherapy 4	62	Not done		

LM, lymphatic malformation; LVM, lymphaticovenous malformation; VM, venous malformation; PIK3CA, phosphatidylinositol-4,5-bisphosphate 3-kinase catalytic subunit alpha. Numbers after intervention types indicate the number of procedures performed. Lesional tissue biopsy was performed for genetic analysis when clinically indicated

### Treatment patterns and response

**Fig. 1:**
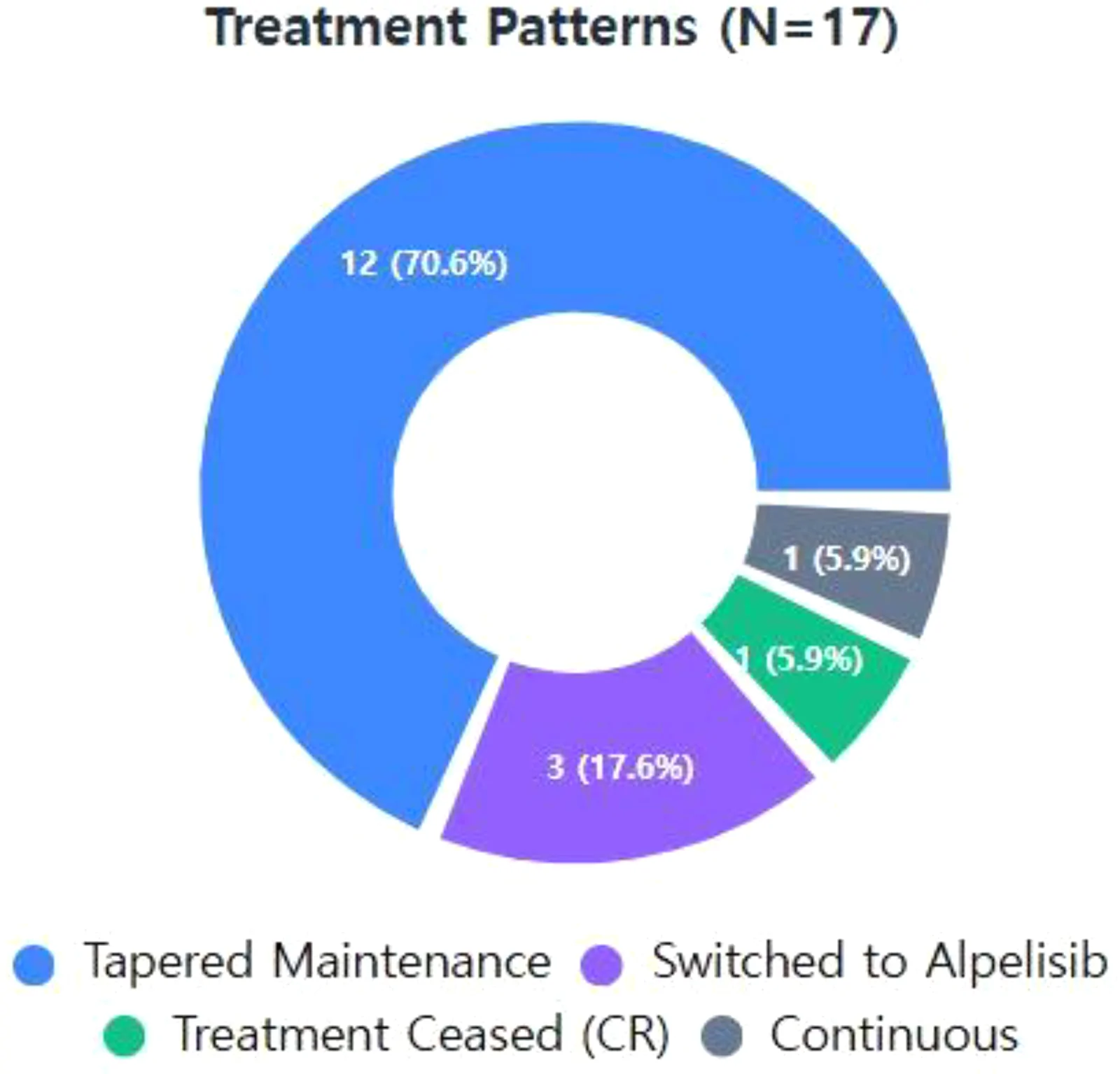
Treatment patterns in patients with vascular anomalies (*N* = 17)

During follow-up of sirolimus therapy, 12 patients (70.6%) successfully underwent medication tapering without disease progression. One patient experienced relapse after complete discontinuation but responded well to reinitiation. Three patients (17.6%) were switched to alpelisib due to suboptimal response (*n* = 1), recurrent infections (*n* = 1), or cellulitis (*n* = 1). One patient ceased treatment after achieving a complete response (CR) without recurrence (Fig. 1).

**Fig. 2:**
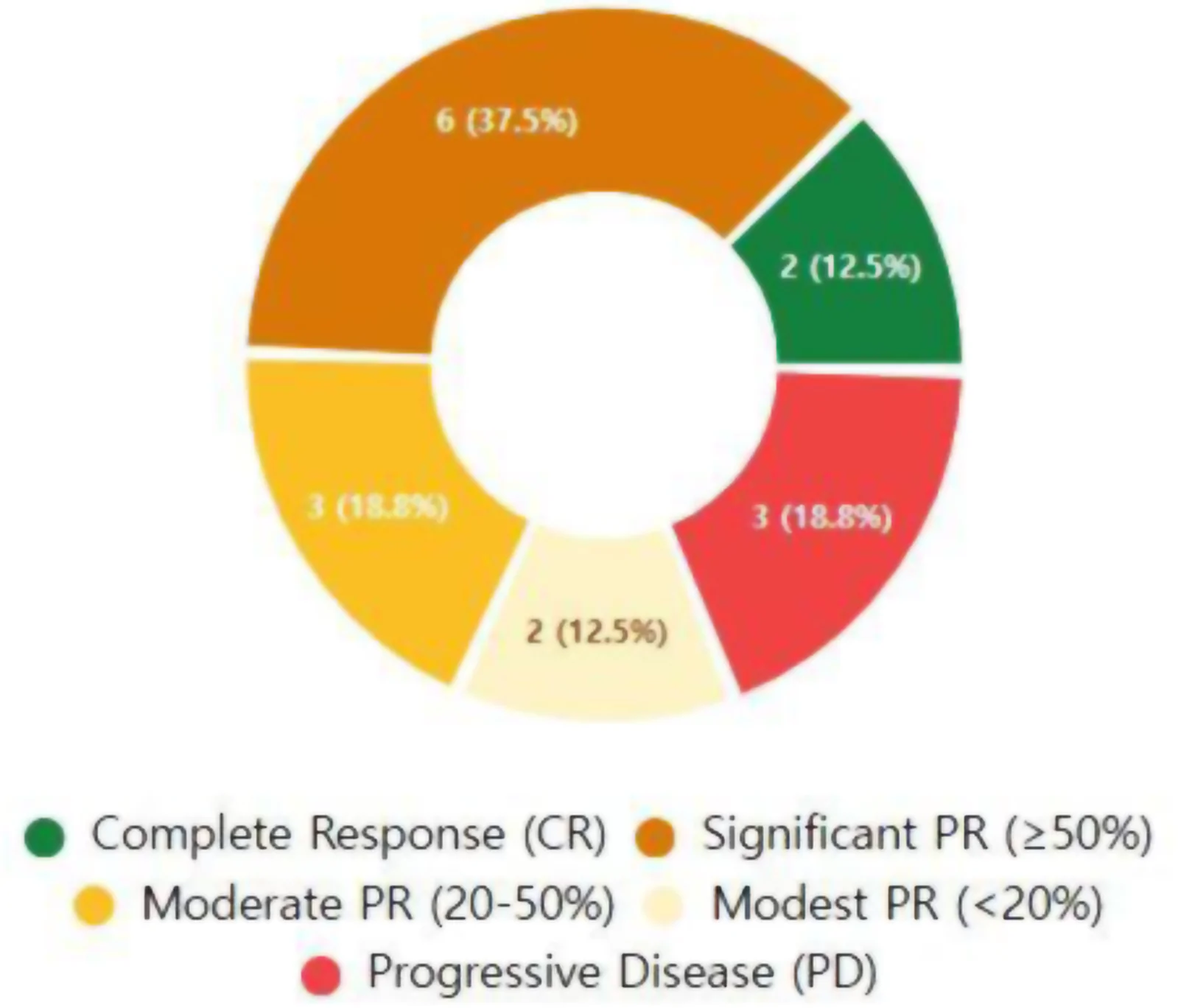
Overall treatment response based on volumetric analysis (*N* = 16). One patient was excluded from this volumetric analysis due to the absence of baseline body surface area (BSA) data required for normalization

The overall response rate (ORR) was 81.3% (13/16 evaluated), with 2 patients achieving CR (12.5%) and 11 achieving a partial response (PR, 68.8%). Among those with PR, 6 demonstrated significant (≥ 50%), 3 moderate (20–50%), and 2 modest (< 20%) volume reduction. Three patients (18.8%) exhibited progressive disease (Fig. 2). The median change in normalized lesion volume was − 45% (IQR: -69% to + 11%), reflecting a consistent trend toward volumetric reduction across the cohort.

**Fig. 3:**
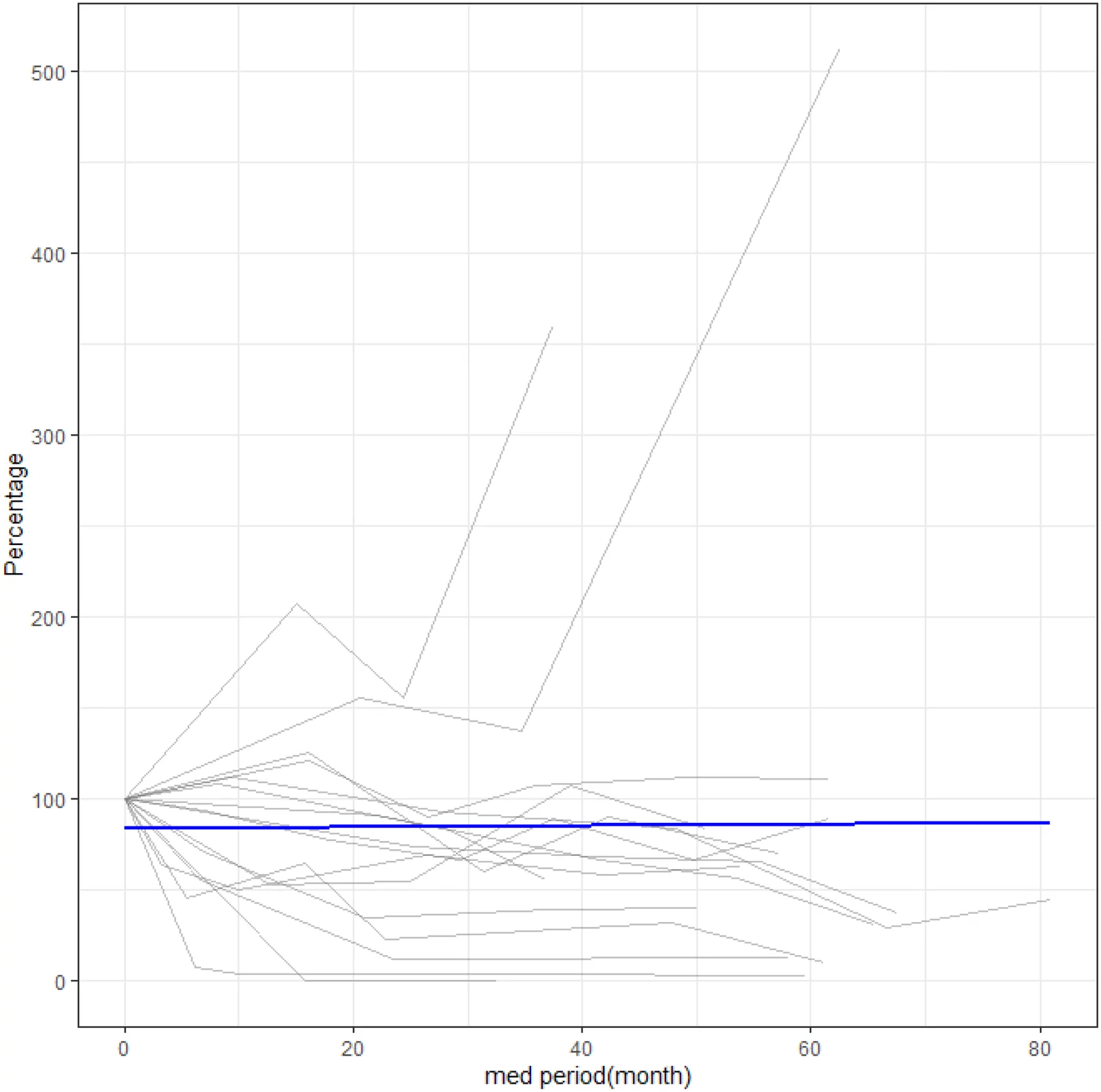
Spaghetti plot of response profiles over time. The y-axis represents percentage change in normalized lesion volume from baseline, and medication duration is expressed in months

**Fig. 4:**
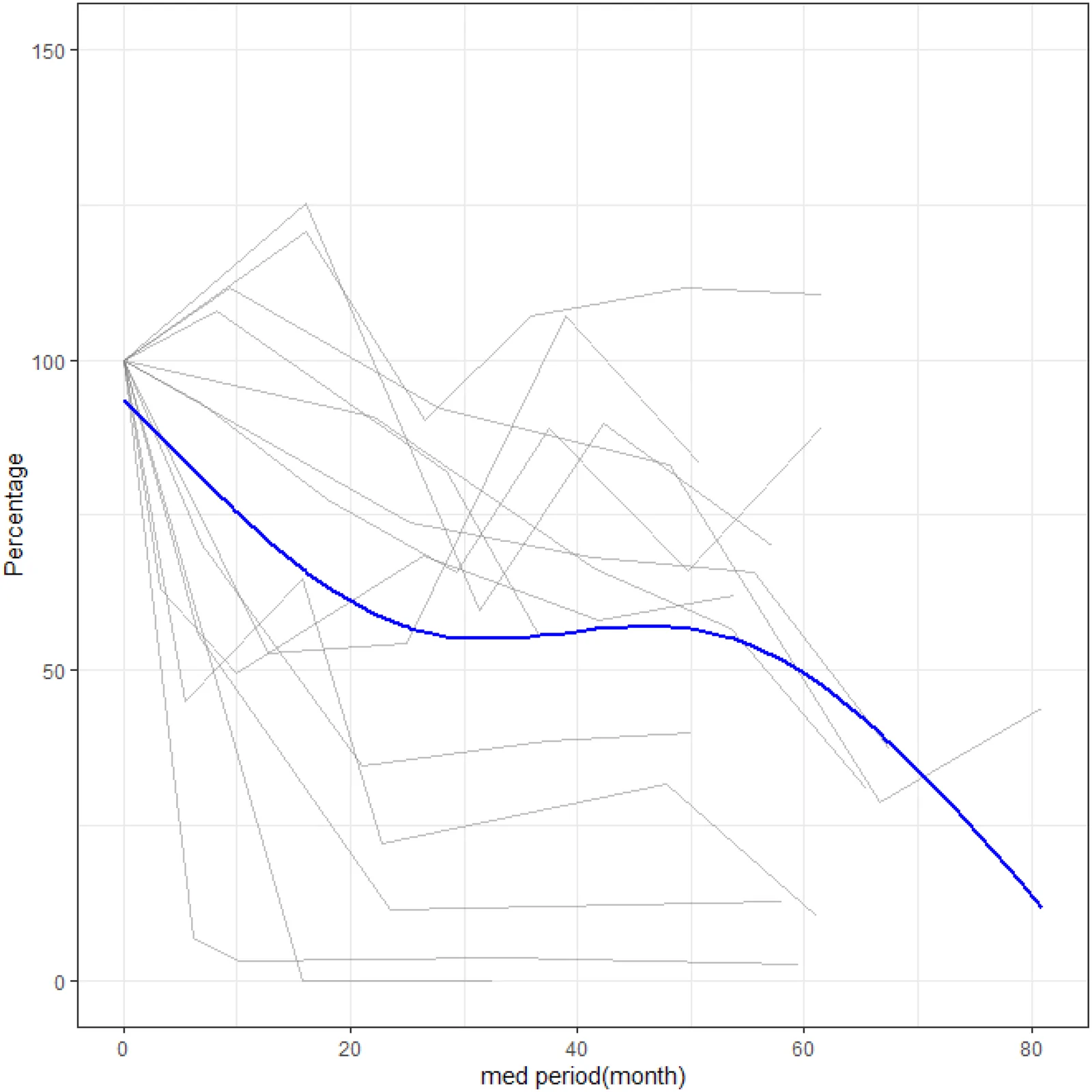
Results from analysis of generalized additive mixed-effects model with random intercept for each subject (*N* = 14 in the sensitivity model excluding patient 16 and the two extreme trajectories). The y-axis represents percentage change in normalized lesion volume from baseline, and medication duration is expressed in months

Figure 3 presents a spaghetti plot illustrating the longitudinal trajectories of normalized lesion volumes for the entire cohort (*N* = 17). The plot demonstrates substantial heterogeneity in individual responses, ranging from near-complete regression to progressive enlargement. Patient 16 was excluded from the normalized volume analysis because baseline BSA data were unavailable. Patients 7 and 8 demonstrated marked volumetric increases (259% and 412%, respectively), which disproportionately influenced the overall model fit; therefore, sensitivity analyses were conducted with and without these patients to evaluate the robustness of the observed trends. An initial linear mixed-effects model did not adequately capture these longitudinal patterns (*p* = 0.9492), likely reflecting non-linearity in treatment trajectories. Therefore, a flexible additive mixed-effects framework was employed. The resulting model (Fig. 4; *N* = 14 after excluding patient 16 and the two extreme trajectories for sensitivity analysis) demonstrated a significant non-linear association between treatment duration and volume change (smooth term F = 18.57, *p* < 0.0001; edf = 2.71). The fitted curve indicates a rapid reduction in lesion volume during the first 12–24 months, followed by relative stabilization. Although a continued decline is visually suggested beyond 60 months, statistical certainty in this extended period is limited by reduced sample size. Overall, the most robust finding is the early therapeutic reduction and subsequent maintenance of treatment gains.

**Fig. 5:**
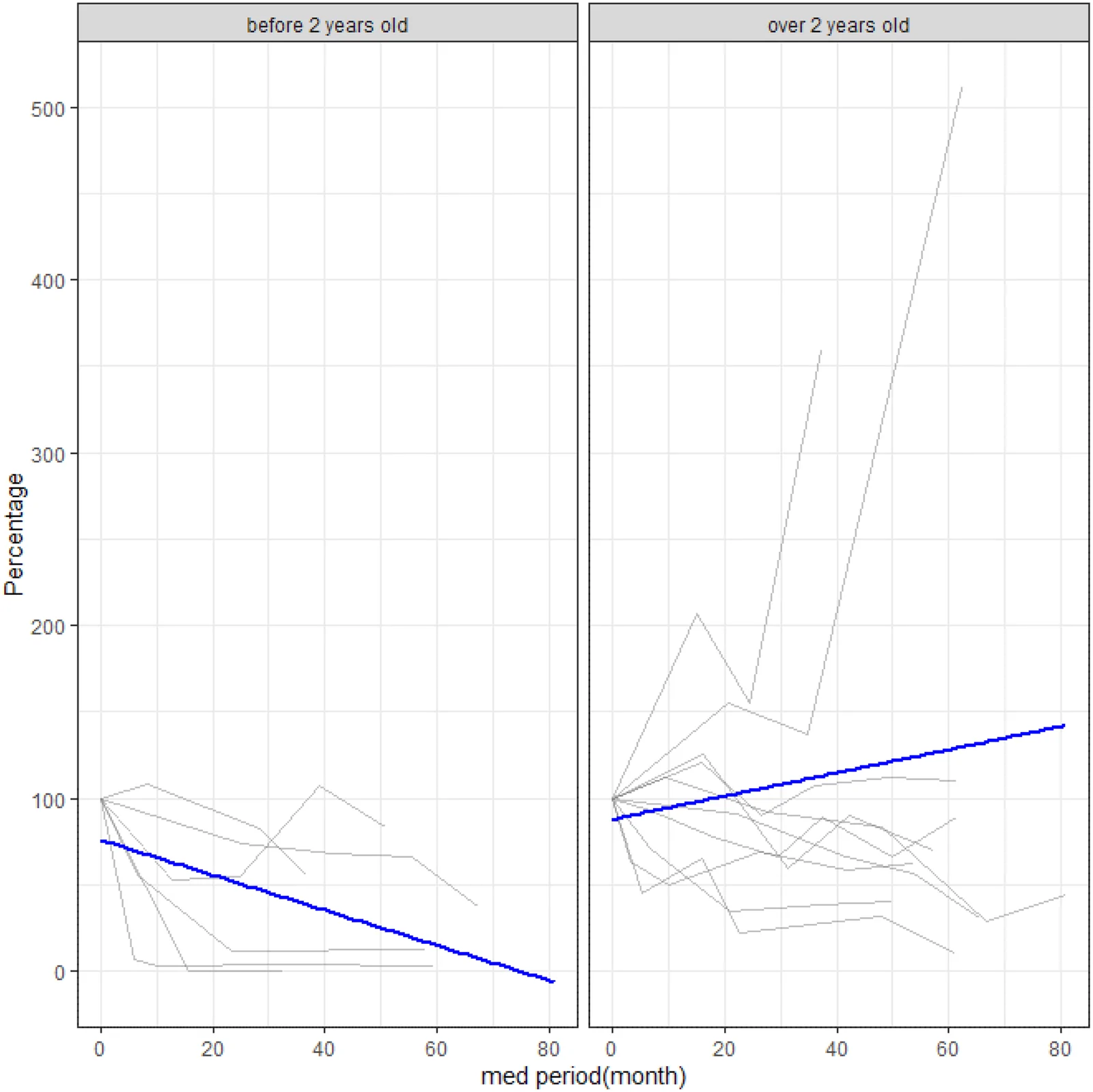
Spaghetti plot of response profiles over time by treatment start before and after the age of two years. The y-axis represents percentage change in normalized lesion volume from baseline, and medication duration is expressed in months

Although no statistically significant difference was observed (interaction *p* = 0.428; Fig. 5), patients treated before 2 years of age demonstrated a more pronounced trend toward volume reduction over time compared with those treated at ≥ 2 years of age (slope = − 1.65 vs. −0.32).

**Fig. 6:**
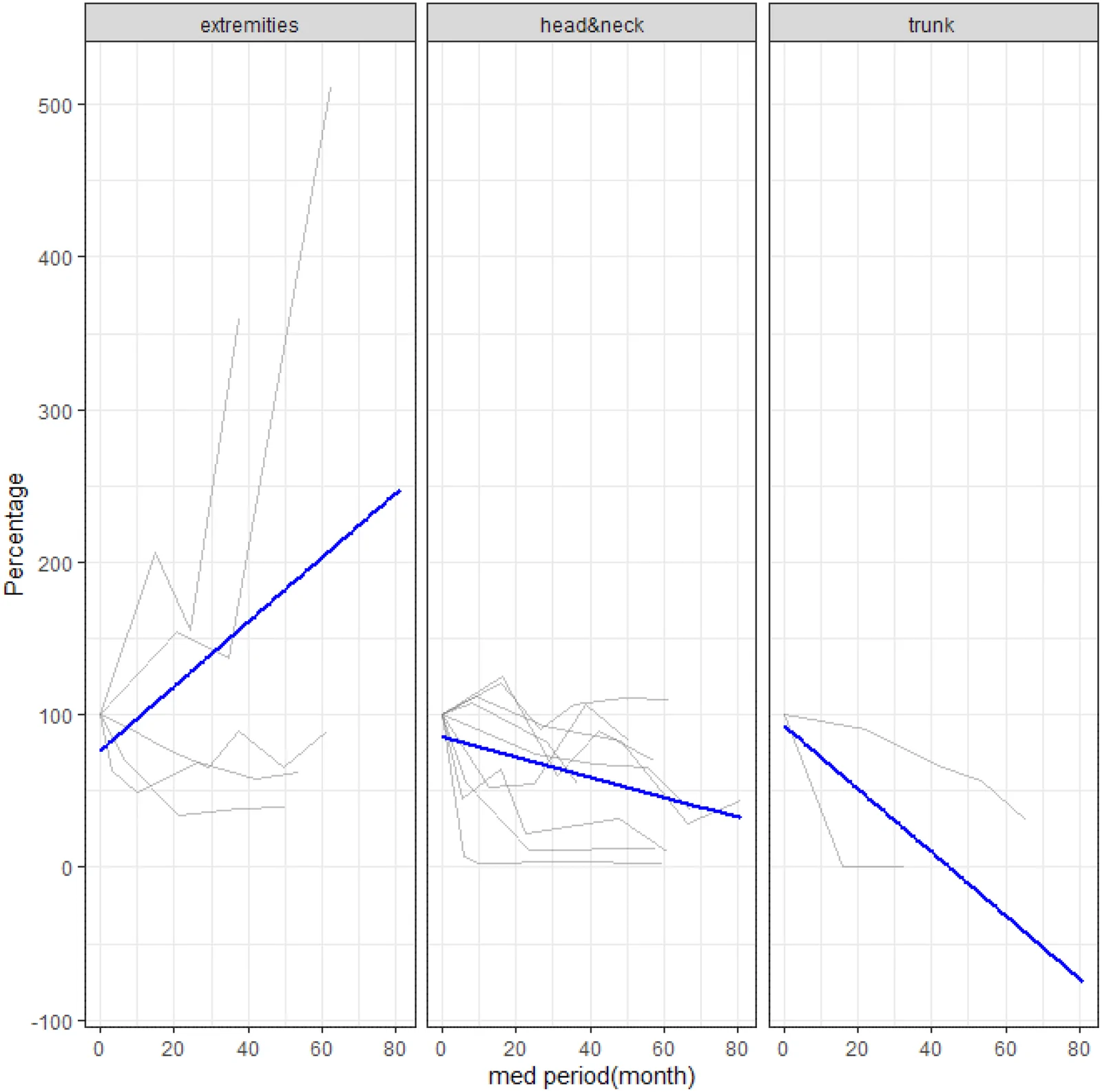
Spaghetti plot of response profiles over time by lesion localization. The y-axis represents percentage change in normalized lesion volume from baseline, and medication duration is expressed in months

Lesion location (head and neck, trunk, extremities) showed no statistically significant differences in treatment response trajectories among groups (interaction *p* = 0.612) (Fig. 6). However, visual examination of the fitted regression lines suggested a trend toward greater volume reduction in patients with head and neck lesions (slope = -1.23) and trunk lesions (slope = -0.87) compared to extremity lesions (slope = + 0.42), though these differences did not reach statistical significance in our limited sample size.

Adverse events during sirolimus treatment were generally mild and manageable. No Grade 3 or higher adverse events related to sirolimus were documented. The most common adverse event was upper respiratory tract infection, occurring in 6 patients (35.3%). Oral ulcers developed in 3 patients (17.6%), and single occurrences of diarrhea, skin rash, and menstrual irregularity were observed (5.9% each). Patient 3’s switch to alpelisib was motivated by both recurrent infections affecting daily activities and suboptimal disease control rather than severe toxicity.

### Response to alpelisib switch

Three patients were switched from sirolimus to alpelisib after 44–48 months of sirolimus treatment. Patient 3 and Patient 16, who had adequate follow-up after the switch (> 12 months), demonstrated clinical improvement with reduction in lesion size on volumetric MRI, showing 42% and 38% decreases, respectively, within 12 months of alpelisib initiation.

## Discussion

This study presents one of the longest follow-up experiences with sirolimus treatment for pediatric vascular anomalies, with a median follow-up of 78 months. Our findings demonstrate that sirolimus maintains a favorable safety profile with long-term use and achieves an overall response rate of 81%, consistent with previously reported short-term studies [8–10]. However, our longitudinal analysis reveals important insights into the temporal dynamics of treatment response and identifies clinical scenarios where alternative therapeutic strategies may be considered.

In our study, most responding patients demonstrated lesion volume reduction during the initial 12–24 months of treatment, whereas subsequent measurements showed minimal additional change. The initial linear mixed-effects model did not demonstrate a statistically significant trend in continued volume reduction (*p* = 0.949), supporting the use of flexible non-linear modeling. This pattern may indicate that the therapeutic effect of sirolimus reaches a plateau after approximately two years of treatment, with important implications for treatment duration and monitoring strategies.

The biological basis for this plateau effect remains unclear but may reflect several mechanisms. First, sirolimus is primarily cytostatic rather than cytolytic; it suppresses cell proliferation and angiogenesis without directly inducing cell death [12]. Consequently, volume reduction may reach a limit once maximal inhibition of these processes is achieved. Second, feedback activation of compensatory signaling pathways, such as PI3K/AKT reactivation through mTORC2 or receptor tyrosine kinase upregulation, may limit efficacy over time [13, 14]. Finally, chronic vascular anomalies involve secondary structural changes – including fibrosis, adipose accumulation, and lymphatic congestion – that are irreversible by mTOR inhibition once tissue remodeling is established.

These observations support a treatment strategy of intensive monitoring during the first 24 months, followed by consideration of medication tapering in responding patients or switch to alternative agents in those with minimal response. Our tapering protocol, successfully implemented in 71% of patients, allowed for reduced medication exposure while maintaining disease control in most cases.

The heterogeneity in treatment response observed in the spaghetti plots likely reflects diverse molecular pathogenesis and lesion-specific biology. However, because PI3CA mutation analysis was performed in only 41.2% of our cohort, our study cannot establish a definitive genotype-response relationship. Venot et al. reported that specific PIK3CA mutation variants may be associated with differential sensitivity to pathway inhibition [15]. Taken together, these findings provide a rationale for prospective studies that incorporate systematic molecular profiling to define individualized therapeutic strategies for vascular anomalies better.

An important contribution of our study is the demonstration that patients with limited response to long-term sirolimus therapy may benefit from switching to alpelisib, particularly when PIK3CA mutations are identified. Two of three patients who switched to alpelisib achieved substantial clinical improvement after 24–36 months of minimal response to sirolimus. This observation aligns with emerging evidence that direct PI3K inhibition may overcome resistance mechanisms that limit mTOR inhibitor efficacy [16, 17]. Alpelisib, as a PI3Kα-specific inhibitor, directly targets the mutant PIK3CA enzyme and may providde more complete pathway suppression in selected patients [18].

However, alpelisib use is associated with distinct toxicity profiles, most notably hyperglycemia due to inhibition of insulin signaling [19]. Patient 1’s development of elevated HbA1C exemplifies this concern and necessitates careful metabolic monitoring and potentially earlier intervention. The substantially higher cost of alpelisib compared to sirolimus also represents a practical barrier to its widespread use, emphasizing the importance of patient selection and molecular confirmation before switching.

Our findings suggest a cautious clinical framework in which sirolimus remains a preferred first-line agent for complicated vascular anomalies, while switching to alpelisib may be considered after 24–36 months in selected patients with documented PIK3CA mutations and minimal response to adequate sirolimus therapy. Tissue biopsy for molecular analysis may be considered in cases of suboptimal response to guide subsequent therapeutic decisions, but systematic molecular profiling in larger cohorts is required before definitive treatment algorithms can be established.

While our subgroup analyses by lesion location did not reveal statistically significant differences, trends observed in the data merit cautious discussion. Patients with head and neck lesions and trunk lesions appeared to demonstrate better responses compared to extremity lesions. This observation may reflect differences in lesion biology by anatomic site, but it should be interpreted as exploratory because of the small sample size.

The trend toward better outcomes in patients starting treatment before 2 years of age, though not statistically significant in our cohort, aligns with previous suggestions that earlier intervention may prevent progressive expansion and anatomic complications [20]. Prospective studies with larger sample sizes are needed to definitively establish whether age at treatment initiation predicts long-term outcomes.

This study has several limitations, including its retrospective single-center design, small sample size, and heterogeneity of lesion types, which limit generalizability and statistical power. Subgroup analyses by age at initiation and lesion location should therefore be interpreted as exploratory rather than confirmatory. Incomplete PIK3CA mutation analysis restricts robust genotype–phenotype correlations, and the criteria for biopsy were clinically selected rather than standardized. The absence of a control group precludes definitive attribution of volume changes solely to sirolimus, although prior randomized data have demonstrated its superiority over observation [9]. In addition, our initial target sirolimus trough level of 8–12 ng/mL reflected institutional practice during the study period and was slightly higher than the 4–10 ng/mL target used in some recent studies, which have demonstrated efficacy with potentially lower toxicity [21]. Therefore, our findings should not be interpreted as defining an optimal long-term target trough range, and future protocols may need to evaluate lower target levels, particularly during maintenance therapy. Reliance on volumetric imaging may not fully capture clinically meaningful outcomes, and the limited experience with alpelisib, involving only three patients with short follow-up, prevents definitive conclusions regarding optimal switching strategies and long-term safety and efficacy. Larger multicenter prospective studies incorporating systematic molecular profiling are standardized dosing strategies are needed to establish evidence-based guidelines for managing sirolimus-resistant cases.

## Conclusion

In conclusion, sirolimus demonstrated sustained long-term efficacy and acceptable safety in pediatric patients with complicated vascular anomalies. In patients with limited response, particularly those harboring PIK3CA mutations, transition to PI3K-targeted therapy may be considered. Future multicenter prospective studies incorporating systematic molecular profiling are needed to refine long-term treatment algorithms.

## Data Availability

The datasets generated and/or analyzed during the current study are not publicly available due to patient privacy and institutional regulations but are available from the corresponding author on reasonable request.

## Abbreviations

BSA: Body surface area
CR: Complete response
CTCAE: Common Terminology Criteria for Adverse Events
GAMM: Generalized additive mixed-effects model
LM: Lymphatic malformation
LVM: Lymphaticovenous malformation
MRI: Magnetic resonance imaging
ORR: Overall response rate
PD: Progressive disease
PR: Partial response
VAs: Vascular anomalies
VM: Venous malformation
mTOR: Mechanistic target of rapamycin
PIK3CA: Phosphatidylinositol-4,5-bisphosphate 3-kinase catalytic subunit alpha
